# Effectiveness of third vaccine dose for coronavirus disease 2019 during the Omicron variant pandemic: a prospective observational study in Japan

**DOI:** 10.1038/s41598-022-17990-7

**Published:** 2022-08-10

**Authors:** Tetsuya Akaishi, Shigeki Kushimoto, Yukio Katori, Noriko Sugawara, Hiroshi Egusa, Kaoru Igarashi, Motoo Fujita, Shigeo Kure, Shin Takayama, Michiaki Abe, Akiko Kikuchi, Minoru Ohsawa, Kota Ishizawa, Yoshiko Abe, Hiroyuki Imai, Yohei Inaba, Yoko Iwamatsu-Kobayashi, Takashi Nishioka, Ko Onodera, Tadashi Ishii

**Affiliations:** 1 Department of Education and Support for Regional Medicine, Tohoku University, Seiryo-machi 1-1, Aoba-ku, Sendai, Miyagi 980-8574 Japan; 2 Division of Emergency and Critical Care Medicine, Tohoku University Graduate School of Medicine, Sendai, Japan; 3 Department of Otolaryngology-Head and Neck Surgery, Tohoku University Graduate School of Medicine, Sendai, Japan; 4 Department of Pediatrics, Tohoku University Graduate School of Medicine, Sendai, Japan; 5 Division of Molecular and Regenerative Prosthodontics, Tohoku University Graduate School of Dentistry, Sendai, Japan; 6 Division of Craniofacial Anomalies, Tohoku University Graduate School of Dentistry, Sendai, Japan; 7 Department of Emergency and Critical Care Medicine, Tohoku University Hospital, Sendai, Japan; 8 Clinical Skills Laboratory, Tohoku University School of Medicine, Sendai, Japan; 9 Course of Radiological Technology, Health Sciences, Tohoku University Graduate School of Medicine, Sendai, Japan; 10 Department of Dental Infection Control, Tohoku University Hospital, Sendai, Japan; 11 Liaison Center for Innovative Dentistry, Tohoku University Graduate School of Dentistry, Sendai, Japan

**Keywords:** Medical research, Infectious diseases

## Abstract

The administration of a third booster dose of messenger ribonucleic acid (mRNA) vaccines against coronavirus disease 2019 (COVID-19) has progressed worldwide. Since January 2022, Japan has faced a nationwide outbreak caused by the Omicron variant, which occurred simultaneously with the progression of mass vaccination with the third booster dose. Therefore, this study evaluated the effectiveness of the third dose of vaccine by reverse transcription-polymerase chain reaction (RT-PCR) test using nasopharyngeal swab samples from adults aged ≥ 18 years tested after having close contact with COVID-19 cases between January and May 2022. Participants who completed only one dose were excluded from the study. Among the 928 enrolled participants, 139 had never been vaccinated, 609 had completed two doses, 180 had completed three doses before the swab test, and the overall RT-PCR test positivity rate in each group was 48.9%, 46.0%, and 32.2%, respectively. The vaccine effectiveness of the third dose to prevent infection after close contact was approximately 40% (95% confidence interval: 20–60%), which was the highest at 10–70 days after receiving the third dose. In conclusion, the effectiveness of the three-dose mRNA COVID-19 vaccine after close contact during the Omicron outbreak is approximately 40%.

## Introduction

Coronavirus disease 2019 (COVID-19), caused by severe acute respiratory syndrome coronavirus 2 (SARS-CoV-2), remains a worldwide public health concern in 2022^1^. More than half of the world’s population has completed two doses of messenger ribonucleic acid (mRNA) vaccines against the virus by the end of 2021^2^, and the third dose of the vaccine is currently underway worldwide. Before the worldwide outbreak of the B.1.1.529 (Omicron) variant at the end of 2021, two doses of COVID-19 vaccines were confirmed to be more than 70% effective in suppressing the infection by conventional variants such as B.1.1.7 (Alpha) or B.1.617.2 (Delta)^3–5^. The vaccine effectiveness was estimated to be maintained up to 6 months from the second dose^6^. In Japan, the sixth nationwide wave of infection by the Omicron variant occurred during the progress of the third vaccination for the nations, which was initiated in December 2021. Previous studies evaluating the effectiveness of a third vaccine dose revealed favorable results in suppressing COVID-19 infection and preventing COVID-19-associated hospitalization effectively^7–10^. These previous studies agreed on the importance of receiving a third booster vaccine dose, especially during the Omicron-predominance period, as the two-dose vaccine effectiveness may be significantly reduced against the highly transmissible variant^7^. Meanwhile, most parts of the study periods of the previous studies included seasons before the Omicron-predominant period, and the exact three-dose vaccine effectiveness against infection during the Omicron-predominant period remains largely unevaluated. More real-world effectiveness data regarding the third vaccine dose during the outbreak of this highly transmissible variant in multiple regions of the world are required. In this study, we investigated the effectiveness of a three-dose mRNA vaccine against COVID-19 infection after close contact with COVID-19 cases during the Omicron-predominant period in Japan.

## Methods

### Participants

**Figure 1:**
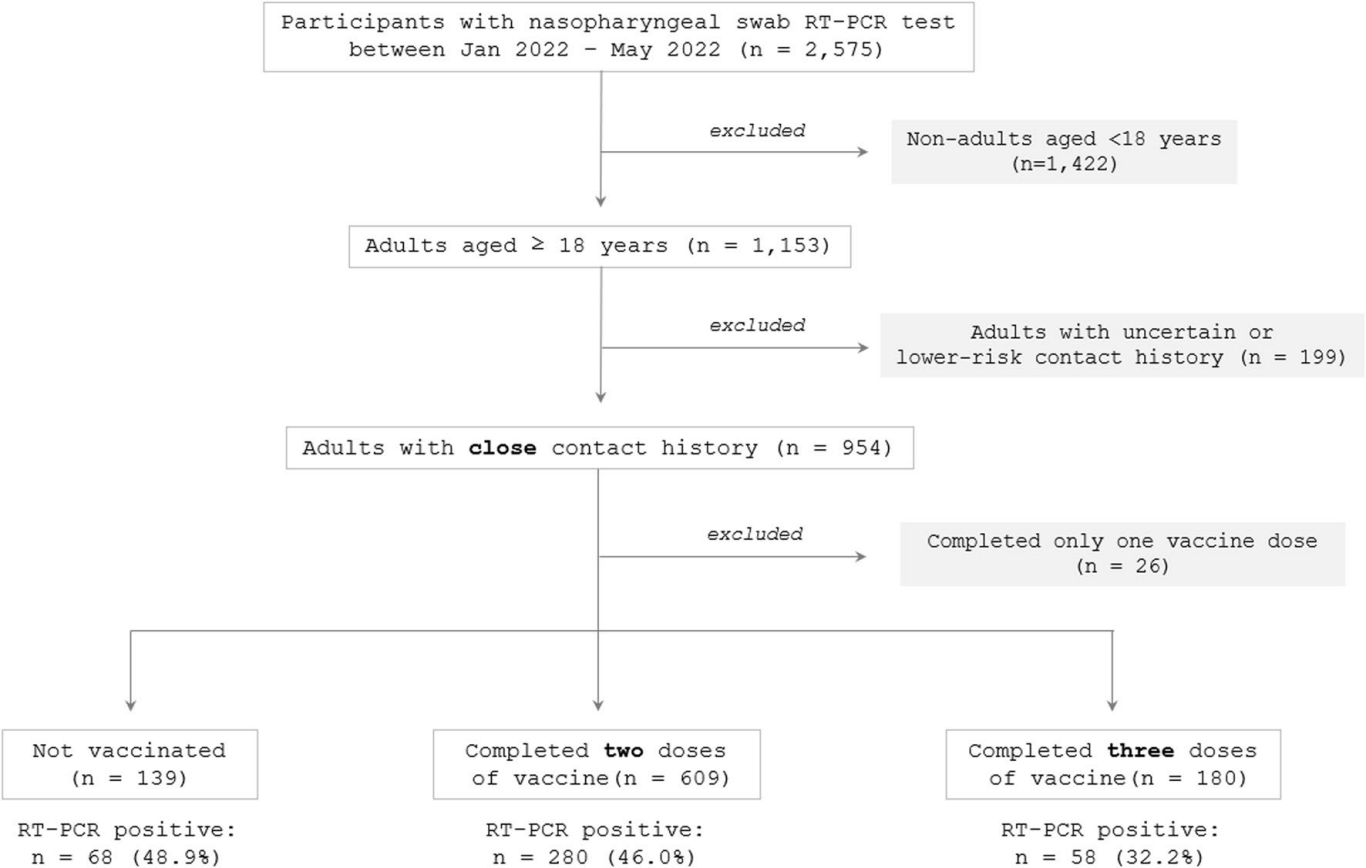
Flow diagram of the study design. Among the overall individuals tested by reverse transcription-polymerase chain reaction (RT-PCR) test using nasopharyngeal swab samples at a large screening test center in Japan between January and May 2022, (1) adults aged < 18 years, (2) those without a certain contact history, (3) those who had completed only one vaccine dose, and (4) those who were less than 7 days after the last vaccination were excluded. Consequently, 767 adults were eligible for subsequent analyses.

This study enrolled adults aged ≥ 18 years living in Miyagi Prefecture who had a history of recent close (high-risk) contact with COVID-19 cases and had provided their nasopharyngeal swab specimens at a drive-through outpatient clinic for the testing of COVID-19 (Tohoku University Medical Office) at a location away from Tohoku University Hospital in Sendai City, Japan, managed by the local governments and Tohoku University, between January and May 2022. This period corresponded to the sixth nationwide wave of the COVID-19 outbreak, exclusively caused by the Omicron variant. During the study period, a sampling test of the viral genome revealed that more than 99% of the infections in the locality were caused by the Omicron variant. Because the main objective of this study was to evaluate vaccine effectiveness in those who had completed three vaccine doses (three-dose group) compared with those who were not vaccinated (no-vaccine group) or had completed two doses (two-dose group), those who had completed only the first vaccine dose (one-dose group) at the time of the nasopharyngeal swab test were excluded from subsequent analyses. This study was conducted before the fourth dose of COVID-19 mRNA vaccines became available in Japan. A flowchart of the study design is shown in Fig. 1.

### Evaluated variables

From these tested individuals, information regarding the demographics (age and sex), detailed situation of the contact, vaccine completion status (number of completed vaccine doses and manufacturer of the vaccines), elapsed time from the last vaccination at the time of swab test, and results of nasopharyngeal swab reverse transcription-polymerase chain reaction (RT-PCR) test for SARS-CoV-2 were collected. The timing of nasopharyngeal swab sampling in most enrolled cases was scheduled 4–5 days after contact with COVID-19 cases. Individuals who had already passed more than 14 days from the last contact history were not tested at the testing center. To evaluate the effectiveness of the vaccine against COVID-19-associated symptoms 4–5 days after the infection, the presence of symptoms including cough, dyspnea, fatigue and a body temperature ≥ 37.5 ℃ was recorded at the time the PCR swab was taken.

### RT-PCR testing

To detect the virus in the sampled swab specimen, RT-PCR was performed to detect the viral nucleocapsid protein set no. 2 (N2) gene. A primer/probe set designed by the National Institute of Infectious Diseases in Japan (NIID_2019-nCoV_N_F2, R2, and P2) was used^11^. The details of the thermal cycling conditions have been previously reported^12^.

### Closeness of the contact

The closeness of contact with COVID-19 cases was judged using the criteria defined by the government. More specifically, fulfillment of all of the following four criteria was considered to be a close contact history: (1) contact with a patient with COVID-19 from 2 to 14 days after the onset of symptoms or positive RT-PCR test results, (2) not wearing masks, (3) contact involving < 1 m distance, and (4) ≥ 15 min of contact. All other contact patterns with patients with COVID-19 were regarded as lower-risk contacts. The closeness of the contact in each of the tested individuals was assessed in advance before the RT-PCR test by the local government staff in public health centers.

### Statistical analysis

The distributions of non-normally distributed variables were described as the median and interquartile range (IQR; 25–75 percentiles). Comparisons of non-normally distributed variables between the two groups were performed using the Mann–Whitney U test, and those between the groups were performed using the Kruskal–Wallis test, followed by the Scheffé post-hoc test. The RT-PCR test positivity rate was used as the marker of the risk of infection in each subgroup, and the rates between those who were not vaccinated (zero-dose group), those who had completed only two doses (two-dose group), and those who had completed all third doses (three-dose group) were compared using the chi-square test. Risk ratios (RR) and 95% confidence intervals (CI) for RT-PCR test-positive participants between those with no vaccination and those who had completed the third booster vaccination were also evaluated. RR was calculated as the risk of infection in the three-dose group divided by the risk in the no-vaccine group. Vaccine effectiveness (%) and 95% CI were estimated as $\left(1-RR\right)\times 100$. Sample size calculation was performed before performing the chi-square test, which revealed a required sample size of n = 32 in each group for a large effect size of φ = 0.50, $\alpha$ = 0.05, and power (i.e., $1-\beta$) = 0.80. Statistical significance was set at *P* < 0.05. Adjustment for multiple testing was not performed because of the nature of the subgroup analyses in this study. To visually confirm the relationship between the elapsed time from the last vaccination and RT-PCR test-positivity rate in the two- and three-dose groups, the rolling average (± 5 days) of the RT-PCR test positivity rate in these groups was depicted. Statistical comparisons and sample size calculations were performed using R Statistical Software (version 4.0.5; R Foundation, Vienna, Austria).

### Ethical approval

All methods were performed in accordance with relevant guidelines and regulations. All study protocols were approved by the institutional review board of the Tohoku University Graduate School of Medicine (approval number: 2020-1-535). Informed consent was obtained from all the participants.

## Results

### Background of the participants

A total of 928 consecutive adults aged ≥ 18 years (413 men and 515 women) who fulfilled the following criteria were enrolled: (1) had close contact with patients with COVID-19 between January and May 2022 and were sampled for nasopharyngeal swabs at the above-described testing center, (2) were vaccinated 0, 2, or 3 times, and (3) provided detailed contact history and vaccine completion status. 858 (92.5%) were household contacts and 70 (7.5%) were non-household contacts. As for the vaccine completion status, 139 (15.0%) belonged to the zero-dose group, 609 (65.6%) to the two-dose group, and 180 (19.4%) to the three-dose group (Table 1). The median (IQR) age in each group was 35 (31–41), 37 (32–43), and 42 (34–65) years. The age in the three-dose group was significantly higher than that in the zero- and two-dose groups (*P* < 0.0001). This was because older adults aged ≥ 65 years are given priority to receive vaccinations in Japan. The mean (IQR) elapsed days from the last vaccination was 158 days (125–189) in the two-dose group and 36 days (20–59) in the three-dose group, which was significantly longer in the two-dose group (*P* < 0.0001). The overall RT-PCR test positivity rates in each group were 48.9% (n = 68/139), 46.0% (n = 280/609), and 32.2% (n = 58/180). Regarding the type of the third mRNA vaccine dose in the three-dose group, 128 received the BNT162b2 mRNA vaccine (Pfizer/BioNTech) and 36 received the mRNA-1273 vaccine (Moderna), and the vaccine type used was unknown in 16 participants. The RT-PCR test positivity rate in the BNT162b2 mRNA vaccine group was 28.1% (n = 36/128) and that in the mRNA-1273 vaccine group was 41.7% (n = 15/36). The rate was not significantly different between the two manufacturers (p = 0.1210).

**Table 1:** Sensitivity analysis of RT-PCR test-positive rate after close contact by the elapsed days from the last vaccination and age groups.

Completed vaccine doses	Elapsed days from the last vaccination	All RT-PCR tested participants (n)	RT-PCR test-positive (n)	RT-PCR test positivity rate (%; 95% CI)	RR (95% CI) vs. zero dose group	RR (95% CI) vs. two doses group
Not vaccinated	Total	139	68	48.9% (40.8–57.2)	Reference (1.0)	–
	(18–64 years old)	135	68	50.4% (42.0–58.7)	–	–
	(≥ 65 years old)	4	0	–	–	–
Two doses (total)	7–90 days	40	15	37.5% (24.2–53.0)	0.77 (0.50–1.18)	Reference (1.0)
	91–180 days	352	164	46.6% (41.4–51.8)	0.95 (0.78–1.17)	Reference (1.0)
	≥ 181 days	201	93	46.3% (39.5–53.2)	0.95 (0.76–1.19)	–
(18–64 years old)	7–90 days	39	15	38.5% (24.9–54.1)	0.79 (0.51–1.21)	Reference (1.0)
	91–180 days	351	163	46.4% (41.3–51.7)	0.95 (0.77–1.16)	Reference (1.0)
(≥ 65 years old)	7–90 days	1	0	–	–	–
	91–180 days	1	1	–	–	–
Three doses (total)	7–90 days	150	43	28.7% (22.0–36.4)	0.59 (0.43–0.79)	0.76 (0.48–1.23)
	91–180 days	13	7	53.9% (29.2–76.8)	1.10 (0.65–1.87)	1.16 (0.69–1.94)
	≥ 181 days	None	None	–	–	–
(18–64 years old)	7–90 days	108	35	32.4% (24.3–41.7)	0.66 (0.48–0.91)	0.84 (0.52–1.36)
	91–180 days	11	7	63.6% (35.4–84.8)	1.30 (0.81–2.10)	1.37 (0.87–2.17)
(≥ 65 years old)	7–90 days	42	8	19.1% (10.0–33.3)	0.39 (0.20–0.74)	–
	91–180 days	2	0	–	–	–

RT-PCR test positivity rate after close contact with COVID-19 cases among adults aged ≥ 18 years, stratified by age groups (18–64 years / ≥ 65 years) and elapsed days from the last vaccination (7–90 days, 91–180 days, and ≥ 181 days) are shown.

*CI* confidence interval, *RR* risk ratio, *RT-PCR* reverse transcription polymerase chain reaction.

### Elapsed time from the last vaccination and vaccine effectiveness

**Figure 2:**
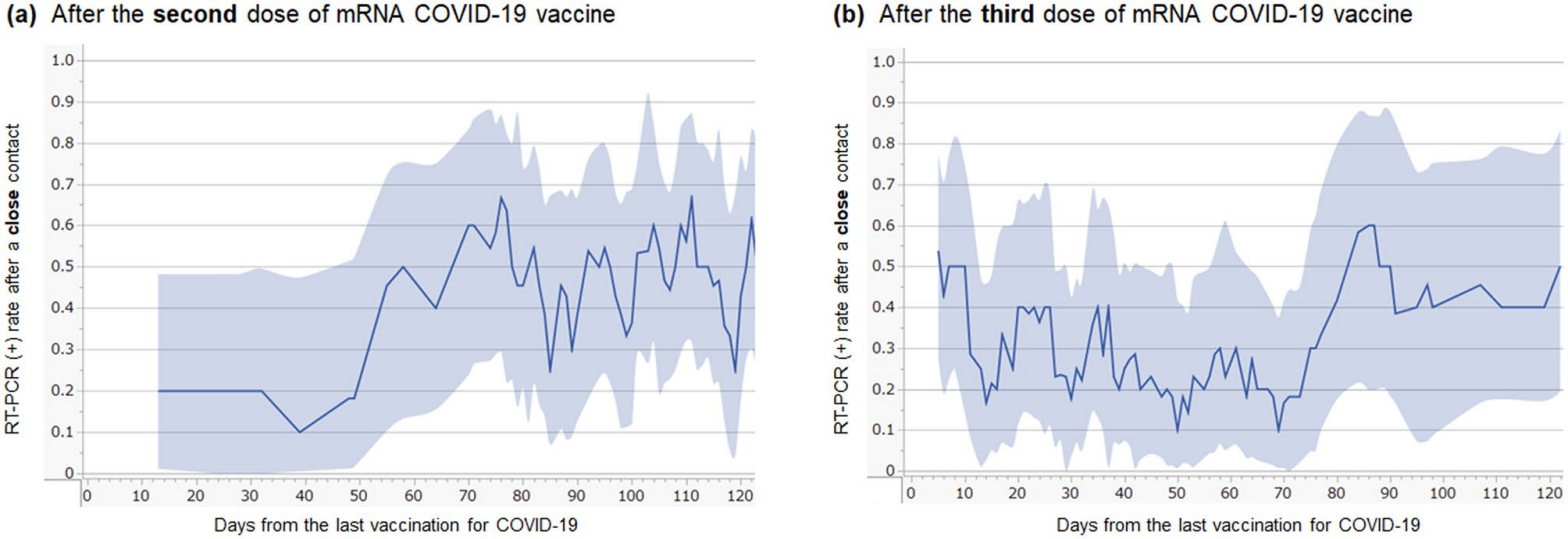
RT-PCR test-positive rate after close contact with COVID-19 cases by the time of the last vaccination. Line graphs for the RT-PCR test-positive rate after close contact with COVID-19 cases, according to the days of the last vaccination in those who completed two (**a**) or three (**b**) doses of mRNA COVID-19 vaccines are shown. The lines represent the rolling average of the test-positive rate within the nearby ± 5 days for each day relapsed from the last vaccination. The blue-filled areas above and below the line graphs represent the 95% confidence interval of the test-positive rate. COVID-19, coronavirus disease 2019; mRNA, messenger RNA; RT-PCR, reverse transcription-polymerase chain reaction.

To evaluate the relationship between the elapsed time from the last vaccination and vaccine effectiveness against infection after the second or third vaccine dose, the rolling average (± 5 days) of the RT-PCR test positivity rate after close contact was obtained for both the two- and three-dose groups (Fig. 2). Vaccine effectiveness in the two-dose group was the highest before 50 days from the last vaccination, whereas that in the three-dose group was the highest between 10 and 70 days after the last vaccination. In both groups, the effectiveness was suggested to gradually weaken thereafter, and more than 90 days after vaccination fell to levels similar to those in the zero-dose group. Based on the result, subgrouping by the elapsed time from the last vaccination in the subsequent analyses was performed with the following three ranges: 7–90 days, 91–180 days, and ≥ 181 days.

### Three-dose vaccine effectiveness against infection

The RT-PCR results for each vaccine completion group are summarized on the right side of Table 1. Because the distribution of age and elapsed time from the last vaccination were significantly different between the two- and three-dose groups, the test-positivity rates were evaluated after stratification by age group (18–64 years / ≥ 65 years) and elapsed time from the last vaccination (7–90 days, 91–180 days, and ≥ 181 days) in each group. The estimated vaccine effectiveness, calculated by $1-RR$, and the 95% CI of the third vaccine dose were approximately 40% (20–60%) at 7–90 days after receiving the third dose when using the zero-dose as the reference group. The significant effectiveness of the third dose was confirmed in both younger (18–64 years) and older adults (≥ 65 years). When the vaccine effectiveness in those who received the third vaccine dose 7–90 days earlier as compared with those who received the second dose more than 180 days earlier but did not receive the third dose yet, the calculated RR (95% CI) was 0.62 (0.46–0.83). This means that those who had already passed more than 180 days after the second dose could expect a 40% decrease in the risk of COVID-19 infection after close contact by receiving a third dose.

### Vaccine effectiveness against symptoms in RT-PCR positive participants

To further evaluate the vaccine effectiveness within 7–90 days from the last vaccination against the development of COVID-19-associated symptoms 4–5 days after the infection, the presence of cough symptoms, feeling of dyspnea, fatigability, and body temperature ≥ 37.5 °C among the RT-PCR test-positive cases was compared between the zero-dose, two-dose, and three-dose groups. The prevalence of each symptom in the three groups is summarized in Table 2. Although three of the four symptoms did not achieve statistical significance because of the small sample sizes, the prevalence of the symptoms was suggested to be slightly lower in the three-dose group than in the zero- or two-dose group.

**Table 2:** COVID-19-associated symptoms in RT-PCR test-positive individuals by the number of completed vaccine doses.

COVID-19-associated symptoms	Zero-dose group (n = 68)	Two-dose group (n = 15)	Three-dose group (n = 43)	Cramer’s V	*P* value
Cough, n (%)	33 (48.5%)	10 (66.7%)	16 (37.2%)	0.179	0.1321
Feeling of dyspnea, n (%)	8 (11.8%)	2 (13.3%)	1 (2.3%)	0.164	0.1828
Fatigability, n (%)	17 (25.0%)	5 (33.3%)	3 (7.0%)	0.241	0.0256
BT ≥ 37.5℃, n (%)	8 (11.8%)	2 (13.3%)	1 (2.3%)	0.164	0.1828

The prevalence of COVID-19-associated symptoms among RT-PCR test-positive individuals who were not vaccinated or within 7–90 days after the second or third vaccine dose during the Omicron outbreak in Japan are listed. Although three of the four symptoms did not reach statistical significance, the prevalence of all symptoms was slightly lower in the three-dose group than that in the two- or zero-dose group. *P*-values were obtained using the chi-square test.

*BT* body temperature, *COVID-19* coronavirus disease 2019, *RT-PCR* reverse transcription-polymerase chain reaction.

## Discussion

This study evaluated the effectiveness of the third booster dose of COVID-19 mRNA vaccines against infection and the development of COVID-19-associated symptoms 4–5 days after the infection during the Omicron variant pandemic in Japan. The results indicated that receiving a third vaccine dose conferred protection comparable with that expected after the second dose. Furthermore, the prevalence of COVID-19-associated symptoms was slightly lower in the three-dose group than in the zero-dose group. The estimated effectiveness of the third-dose vaccine against infection after close (high-risk) contact was approximately 40% (95% CI: 20–60%). This decrease seems modest compared with the previously reported vaccine effectiveness before the Omicron-predominant period. Although the evaluated outcomes were different between the present and previous studies, this may imply that the vaccine effectiveness might have decreased with the Omicron variant compared with other conventional variants before Omicron. A recent study showed a similar finding, indicating that the effectiveness of mRNA COVID-19 vaccines may have decreased against the Omicron variant compared with the previous variants with the estimated adjusted odds ratio of 0.34 (95% CI: 0.32–0.36)^13^. For reference, our previous data indicated that the two-dose vaccine effectiveness against infection after close contact before the Omicron-predominant period would be approximately 70%, supporting the possibility of decreased vaccine effectiveness against infection by the Omicron variant^3^. Another notable finding of this study was that the highest vaccine effectiveness against the Omicron variant was maintained 10–70 days after the last vaccination but gradually decreased thereafter and became almost indistinguishable by 90 days after receiving the second or third vaccination. Based on these, those who had received only two doses but hesitated to take the third dose would benefit from taking it swiftly because the pandemic is still ongoing. Taking the third dose would be effective not only against infection but also against becoming severe. A recent report implied that the third dose of the BNT162b2 mRNA vaccine effectively prevented the patients from resulting in severe COVID-19-related outcomes compared with the patients who had received only two doses^14^. Another recent study reported that three doses of BNT162b2 induced substantially higher neutralization capability against the Omicron variant than that of two doses of the vaccine^15^. Although the severity of the disease may be generally mild with the Omicron variant, many patients would develop critical conditions even with this variant. Therefore, a third dose is especially needed in individuals with predisposing risks to become severe, such as those with older age, male sex, obesity, and some medical histories, including hypertension, pulmonary diseases, and diabetes^16–20^.

This study had several limitations. First, the number of participants in the groups of individuals who had never been vaccinated or completed the third booster vaccination was relatively small. Consequently, the estimated 95% CI of the risk ratio in each subgroup, stratified by age group and elapsed time from the last vaccination, inevitably increased. Further studies with larger numbers of participants are needed to achieve a narrower estimation range for the effectiveness of the third booster vaccination. Second, this study did not evaluate important clinical and demographic data, such as the existence of previous comorbidities, socioeconomic profiles, and subsequent clinical course (i.e., hospitalization and critical conditions). Consequently, this study could not perform additional sensitivity analysis regarding previous comorbidities or estimate vaccine effectiveness against hospitalization or becoming severe. Lastly, not all RT-PCR test-positive participants were checked with genome sequence analysis or variant-specific PCR tests to confirm that they were infected by the Omicron variant. Therefore, whether all the enrolled participants had truly contacted COVID-19 cases with the Omicron variant remains undetermined.

## Conclusions

The estimated three-dose vaccine effectiveness against infection after close contact with COVID-19 cases during the Omicron-predominant period was approximately 40% (95% CI 20–60%), which could be lower than before the emergence of this highly transmissible variant. The third vaccine dose was suggested to slightly decrease the risk of developing COVID-19-associated symptoms 4–5 days after infection. The vaccine effectiveness of the third dose during the Omicron outbreak was the highest within 10–70 days after receiving the second or third vaccine dose, but the effectiveness became uncertain more than 90 days after vaccination. Individuals who completed only two doses and hesitated to receive the third dose would benefit from swift administration of the third dose, especially those with predisposing factors to become severe.

## Supplementary Information

Supplementary Information.

## Data Availability

The whole original data that support the findings of the present study are presented in.
